# Evidence for socially influenced and potentially actively coordinated cooperation by bumblebees

**DOI:** 10.1098/rspb.2024.0055

**Published:** 2024-05-01

**Authors:** Olli J. Loukola, Anna Antinoja, Kaarle Mäkelä, Janette Arppi, Fei Peng, Cwyn Solvi

**Affiliations:** ^1^ Ecology and Genetics Research Unit, University of Oulu, Oulu, 90014, Finland; ^2^ Biology Centre of the Czech Academy of Sciences, Institute of Entomology, Faculty of Science, University of South Bohemia, Branisovska 31, 37005, Czech Republic; ^3^ Department of Psychology, School of Public Health, Southern Medical University, Guangzhou, 510515, People's Republic of China; ^4^ Guangdong-Hong Kong-Macao Greater Bay Area Center for Brain Science and Brain-Inspired Intelligence, Southern Medical University, Guangzhou, 510515, People's Republic of China

**Keywords:** bumblebees, collaboration, comparative cognition, insects, social behaviour

## Abstract

Cooperation is common in animals, yet the specific mechanisms driving collaborative behaviour in different species remain unclear. We investigated the proximate mechanisms underlying the cooperative behaviour of bumblebees in two different tasks, where bees had to simultaneously push a block in an arena or a door at the end of a tunnel for access to reward. In both tasks, when their partner’s entry into the arena/tunnel was delayed, bees took longer to first push the block/door compared with control bees that learned to push alone. In the tunnel task, just before gaining access to reward, bees were more likely to face towards their partner than expected by chance or compared with controls. These results show that bumblebees’ cooperative behaviour is not simply a by-product of individual efforts but is socially influenced. We discuss how bees’ turning behaviours, e.g. turning around before first reaching the door when their partner was delayed and turning back towards the door in response to seeing their partner heading towards the door, suggest the potential for active coordination. However, because these behaviours could also be interpreted as combined responses to social and secondary reinforcement cues, future studies are needed to help clarify whether bumblebees truly use active coordination.

## 1. Introduction

Cooperation is a fundamental aspect of social behaviour, representing instances in which two or more individuals coordinate their actions to achieve outcomes that provide mutual benefits [1]. Cooperation is a widespread phenomenon observed across various species ranging from primates to dolphins [2–5], and fishes to social insects [6–8]. Understanding the cognitive mechanisms underlying animal cooperation has been a growing topic of interest in recent years. Previous studies have primarily focused on cooperative pulling tasks, where animals need to simultaneously pull two ropes or handles in the same direction to obtain a food reward [9]. These studies have examined different measures to assess whether the outcome of cooperation is simply a by-product of animals’ individual efforts, or whether animals comprehend the cooperative nature of the task, and if so to what extent. Measures often include observing whether animals adjust their behaviour to their partner’s presence and actions. For example, do they pull more often when their partner is present, look at their partner more in cooperative situations, wait for a delayed partner or actively recruit a partner [10–27].

Duguid and Melis [5] recently laid out a new categorization of collaborative behaviour that helps distinguish whether animals intentionally coordinate their actions with others. These categories consist of by-product collaboration, socially influenced cooperation, actively coordinated collaboration and collaboration based on shared intentionality. *By-product collaboration* includes joint actions that are not intentionally coordinated but rather occur as a by-product of individual actions or external stimuli. Animals may engage in superficially coordinated actions due to social facilitation or stimulus enhancement without actively intending to collaborate [11,24,27,28]. *Socially influenced cooperation* is where joint actions are influenced by the presence or behaviour of others, but there is no intentional coordination or shared goal. Animals might respond to the actions of others, such as pulling in the presence of a partner, but the collaboration is not based on a mutual understanding of roles or goals [5,28]. *Actively coordinated collaboration* involves animals intentionally coordinating their actions with others to achieve a shared goal. They demonstrate a level of understanding of their partner’s role and may wait for the partner, provide instrumental help or communicate to support the joint action. Species such as chimpanzees (*Pan troglodytes*), bottlenose dolphins (*Tursiops truncatus*), Bornean orangutans (*Pongo pygmaeus*), brown capuchins (*Cebus apella*), Asian elephants (*Elephas maximus*), wolves (*Canis lupus*), spotted hyaenas (*Crocuta crocuta*) and keas (*Nestor notabilis*) have shown evidence of actively coordinated collaboration [11–13,16–18,20,21,29]. *Collaboration based on shared intentionality* involves joint action supported by cognitive skills and motivations to share goals and intentions with others. Animals in this category not only understand their partner’s role but also form shared representations of the shared goal and have a mutual commitment to achieve it together. This form of collaboration is a hallmark of human cooperation and is demonstrated by children from around 3 years old onward (reviewed in [4,30]).

A wider comparative perspective of cooperation requires data from diverse species. Bumblebees (*Bombus* sp.) have not been observed to cooperate in a foraging context [31,32]. However, bumblebees have exhibited various cognitive abilities and processes that are believed to be crucial for cooperative behaviours [33], including social learning [34], flexible problem-solving [35], body awareness [36], object-play [37] and positive emotional states [38]. Although cooperation is common in social insects [7,8], to our knowledge, no direct efforts have been employed towards exploring the proximate cognitive mechanisms of insect cooperation. It is unknown whether any insect understands the role of their partners in cooperative tasks to any degree, or whether instead, insect cooperation is simply a by-product of individual action. To help answer these questions, here, we examined bumblebees’ (*B. terrestris*) behaviour in two different cooperative foraging tasks. Both tasks required bees to enter a designated area and then find and simultaneously push at a specific location in order to gain access to rewarding sugar water. After training, all subjects experienced a test wherein their partner’s access to the designated area was delayed. If bumblebees’ cooperative behaviour is simply a by-product of their individual effort, individual behaviour should remain consistent and unaffected by the presence or actions of their partner. If, however, cooperative behaviour is socially influenced, we should expect adjustments in individual behaviour during the delay test, compared with the training phase and to bees that were not trained to simultaneously push with another bee. Finally, if bees engage in actively coordinated collaboration, they should flexibly respond to the location and activity of their partner in ways that would facilitate cooperation.

## 2. Methods and results

### (a) Subjects

Experiments were conducted at the University of Oulu, Finland. Buff-tailed bumblebees (*B. terrestris*) from Koppert, The Netherlands, were housed in a two-chamber wooden nesting box. For the block-pushing task, the nesting box was connected to a flight arena (*l* = 60 cm, *w* = 45 cm, *h* = 25 cm, with a transparent acrylic top) by a transparent acrylic tunnel (*l* = 25 cm, *w* = 3.5 cm, *h* = 3.5 cm). Individual bees’ access between the nesting box and the arena was controlled with sliding doors in the tunnel. For the tunnel task, the nesting box was connected to two adjacent tunnels (*l* = 30 cm, *w* = 2.5 cm, *h* = 1.5 cm). The two adjacent tunnels were separated by a transparent wall. Individual forager bees were marked with small plastic number tags on their thorax. The experimental set-ups were maintained indoors under standardized light conditions (LED, 2700 K, 230 V AC), with a temperature range of 19–22°C and a 12 h light-dark cycle.

#### (i) Ethical considerations

According to Finnish regulations (Act of the Use of Animals for Experimental Purposes 2006/62 §4), bumblebees are not classified as experimental animals, and therefore, project authorization is not required by the Ministry of Agriculture and Forestry in Finland. The experiments were non-invasive and the stimuli used (water and sugar water) are experienced by bumblebees during their natural foraging life. The bumblebees were cared for on a daily basis by trained and competent staff, which included routine monitoring of welfare.

### (b) Study 1: block-pushing task

#### (i) Methods for block-pushing task

**Figure 1.**
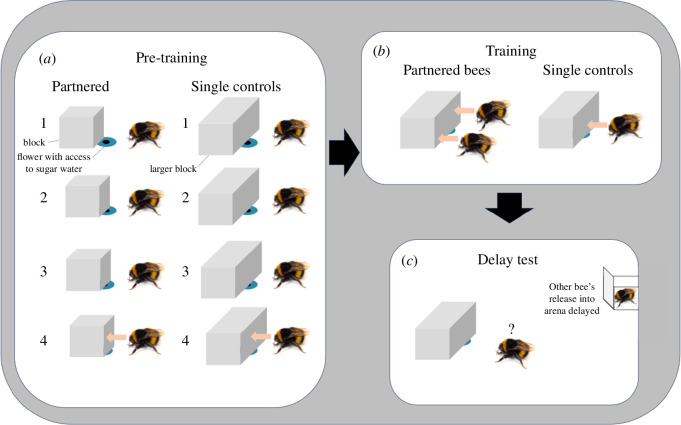
Block-pushing task. Training and testing methods used for the partnered and single control bees. (*a*) Successive steps in pre-training (each step was repeated three times): step 1, bees found reward in a blue flower (blue circle on floor with hole in the middle giving access to sugar water); step 2, 50% of the hole was covered by the block; step 3, 75% of the hole covered; step 4, 100% of the hole covered. (*b*) In training, 100% of the hole was covered by a block, and the bees pushed the block simultaneously (partnered) or alone (single controls) to gain a reward. (*c*) In the delay tests, trained bees from both treatment groups were individually released into the arena with two blocks, each covering a different flower. In experiment 1, one bee was released first and the other bee was released as soon as the first-released bee landed within a body length of the block. In experiment 2, we implemented an additional 30 s delay from when the first-released bee landed next to the block to when the other bee was released. Please see Methods and results for more details.

To investigate whether bumblebee cooperation is a result of individual efforts or is socially influenced, two experiments were conducted using a block-pushing task. In experiment 1, bumblebees were pre-trained in groups to forage from six blue rings (Ø = 1 cm, flower from now on) on the arena floor, spaced 7 cm apart from each other. Twelve partnered bees (4, 4, 4 from three colonies) and 12 single control bees (4, 4, 4 from three colonies) individually underwent a pre-training phase. Partnered bees were trained to move a small white Styrofoam block (1 × 1 × 1 cm) off of the flower in order to gain access to nectar. Single control bees, on the other hand, were pre-trained with a larger block (3 × 2 × 1 cm). Both groups underwent pre-training in a stepwise manner wherein the flower was increasingly covered by the block (figure 1*a*). To discourage the association of reward with one specific location and encourage bees to focus on the task, two randomly selected flowers out of the total six flowers were made accessible in each bout, while the remaining flowers were covered with a thin piece of white plastic, the same material as the floor. Each step (figure 1*a*) was repeated three times before proceeding to the next phase. After the bees successfully completed step 4 with the blocks (see electronic supplementary material, video S1), they were trained either in dyads (partnered, see electronic supplementary material, video S2) or alone (single controls) to push the larger block (3 × 2 × 1 cm) covering the flower (figure 1*b*). The training phase consisted of three bouts. The experimenter controlled each block’s movement with magnets inside the block and underneath the arena floor. This ensured that to gain access to the flower and sugar water, bees were required to push the block simultaneously in the partnered group, while single control bees needed to push alone. The change between the pre-training and training aimed to reinforce the need for cooperation in the partnered group, contrasting with the independent task of single control bees. Pushing was defined as an action where an individual touched the block with their head or front legs (or both) continually for 1 s with their body axis (abdomen to head) perpendicular to the block.

All bees were individually tested in a similar set-up as in training, but with a delay between the two bees’ release into the arena. In each delay test, one bee (first-released bee) was individually released into the arena with two blocks each covering a different flower. Once the first-released bee landed within a body length of a block (signifying to the experimenter that they were engaged in the task), their training partner (for partnered bees) or another control bee (for single controls) was released into the arena (figure 1*c*; electronic supplementary material, video S3). Each bee in both groups experienced two tests, one as the first-released bee and one as the second-released bee. We recorded the time from landing until the first-released bee’s first push, whether the first-released bee’s first push occurred before or after the second-released bee’s first push, and if the first-released bee’s first push occurred when the second-released bee was within one body length of the first-released bee.

In experiment 2, we trained 19 partnered bees (6, 2, 1, 2, 2, 2, 2, 2 from eight colonies) and 17 single control bees (3, 7, 7 from three colonies) similarly as above with several differences. For these bees, the smaller blocks (1.6 × 1.6 × 1.1 cm) and larger blocks (3.2 × 1.6 × 1.1 cm) were white Legos from Billund, Denmark, hollowed out to accommodate a magnet inside. The use of the Legos allowed for easier and quicker cleaning between bouts. To determine whether more training helped bees synchronize better, i.e. shorten the time between release and pushing the block simultaneously, the training phase was repeated 25 times for each bee. Additionally, in the delay test, to determine whether a longer delay time would increase the first-released bee’s time to first push, the delayed bee was released 30 s after the first-released bee landed within a body length of a block. However, we found that the time from the first-released bee’s release to their first push was not any longer in experiment 2 compared with in experiment 1. The lack of increased time to first push was probably due to the large variability across bees in how long it took them to actually leave the antechamber on their own volition after the door was raised.

Because bumblebees normally only live a few weeks, some of the trained bees died of natural causes before finishing the experiment. Five and three partnered bees died or were excluded from the delay test during experiments 1 and 2, respectively. The other bees that were excluded had either stopped flying or engaging with the task just prior to or during the delay test. These eight bees are distinct from the 12 partnered bees in experiment 1 and the 19 partnered bees in experiment 2.

All bouts were recorded using an iPhone 8 (Apple Inc., Cupertino, CA) placed above the arena. Behavioural measurements were obtained through video analysis and are described below. Although group identity was unavoidably obvious for training bouts (one bee = single control and two bees = partnered group), videos were labelled by a non-coder such that the coder was at least initially blind to the group identity for the delay tests (two bees in each group). Experiment 1 was analysed by A.A. (100%) and O.J.L. (5%) while experiment 2 was analysed by K.M. (100%) and O.J.L. (5%). Intraclass correlation (ICC) analysis was employed to assess the agreement among the three observers for the measured variables. The ICC analysis was conducted using R and theICC analysis for intercoder reliability, implemented through the ‘irr’ package (v. 0.84.1) [39]. The mean ICC value was 0.99 (95% CI: 0.98–1) indicating a high level of agreement among the coders. For the statistical analyses, we employed generalized linear mixed models (GLMMs) using the ‘glmmTMB’ function from the package glmmTMB (v. 1.1.8) [40] in R (v. 4.3.0) [41]. GLMMs were applied for the block-pushing task (models 1–3), as described below. Note that experiment number had no effect on any of the response variables within models 1–3 and we therefore visualized the data together.

In model 1, we investigated whether the first-released partnered bees, after landing within a body length of a block, took longer to make their first push in the delay test compared with second-released partnered bees and single control bees. If partnered bees’ coordination in the task was simply a by-product of their individual efforts, the time from landing next to the block until they first pushed the block should be no different between the above groups. But if bees’ coordination was socially influenced, the delay of one’s partner might cause the first-released bees to hesitate and take longer to push. The *time taken to first push on the block* was the response variable. The explanatory variables included the *group* (first-released partnered bees, second-released partnered bees, or single control bees), *experiment* (experiment 1 or experiment 2) and the *test number of first-released bee. Test number of first-released bee* refers to whether a bee acted as a first-released bee in the first delay test or the second delay test. To account for the hierarchical structure of the data, wherein observations were nested within colony/bee pairs/bee individuals, we treated the *colony/bee pair/bee* as a random variable. A Gamma distribution with a logarithmic link function was used as the family.

In model 2, we investigated whether, in the delay tests, the first-released bee pushed the block before the second-released bee. If bees’ coordinated behaviour was a by-product of individual efforts, we should expect the first-released partnered bee’s first push on the block to occur before the second-released bee first pushed. However, if coordination was socially influenced, then we might expect first-released bees to wait to push, possibly to the extent that the second-released bees would have some chance of pushing first. The response variable was *whether the second-released bee pushed before the first-released bee* (yes or no). The explanatory variables included the *group* (first-released partnered bees or single control bees), *experiment* (experiment 1 or experiment 2) and the *test number of the first-released bee*. The *colony/bee pair* was used as a random variable. A binomial distribution with a logarithmic link function was used as the family.

In model 3, we tested whether the second-released bee’s location influenced the first-released bee’s first push on the block. If bees’ efforts were not socially influenced, we should expect that the first-released partnered bees should not wait for the second-released bees before they pushed. But if coordination was socially influenced, bees should be more likely to make their first push only when the second-released bee was close to the block. The response variable was *whether the first-released bee’s first push occurred when their partner was present* (yes or no). We initially included the *group* (first-released partnered bees or single control bees), *experiment* (experiment 1 or experiment 2) and the *test number of the first-released bee* as explanatory variables. However, due to overparametrization and the control bees’ data consisting solely of zeros, we conducted separate analyses for the *experiment* and *test number of the first-released bee* variables. This distinct model, crucially, did not include the *group* as an explanatory factor. The exclusion of the *group* allowed for a focused examination of the specific effects of the *experiment* and *test number of the first-released bee* within the partnered bee group, providing insights into these variables’ impact without confounding factors. These analyses revealed that neither the *experiment* [GLMM: 95% CI = 0.30 (−3.07 to 3.68), *p* = 0.860] nor the *test number of the first-released bee* [GLMM: 95% CI = 1.40 (−0.52 to 3.32), *p* = 0.153] significantly affected whether the first-released bee’s first push occurred when their partner was present. Consequently, we refined the model by excluding these variables and incorporating treatment as the only explanatory variable. A binomial distribution with a logarithmic link function was used as the family. Since the control bees’ data consisted only of zeros, making it impossible to directly fit a GLMM, we employed a permutation test. We permuted the response variable, representing the order of pushing the block or pushing when the partner was present, and fitted the GLMM with the permuted data. This process was repeated 5000 times to create a distribution of model estimates under the null hypothesis of no association. The permutation test allowed us to assess the significance of the observed associations.

#### (ii) Results for block-pushing task

**Figure 2.**
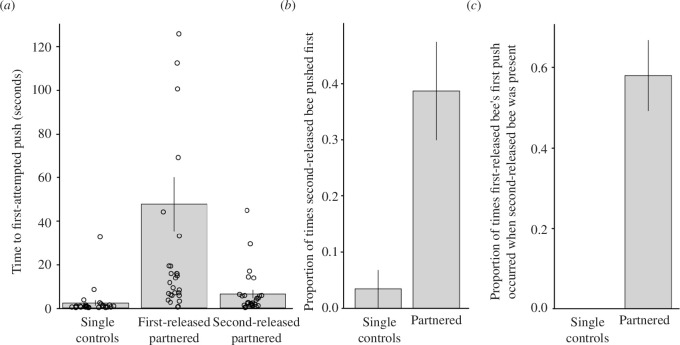
Results of block-pushing task. (*a*) Time, in the delay test, between a bee landing within a body length of the block and their first push on the block. (*b*) The proportion of occurrences in which the second-released bee pushed the block before the first-released bee. (*c*) The proportion of instances in which the first-released bee’s first push occurred when the second-released bee was present at the block. Bars indicate the mean, and vertical lines represent the s.e.m. Open circles in (*a*) represent individual data points. Figures are based on the raw data.

In the delay tests, the time from when a first-released bee landed within a body length of the block until the first-released bee’s first push was longer for partnered bees compared with single controls (model 1; GLMM: 95% CI (back-transformed from the log scale) = 23.92 (12.10–47.31), *n* = 60 bees, *p* < 0.001) and to second-released partnered bees (model 1; GLMM: 95% CI (back-transformed from the log scale) = 6.35 (3.57–11.29), *n* = 60 bees, *p* < 0.001) (figure 2*a*). Neither the *experiment* (GLMM: 95% CI = 1.32 (0.71–2.46), *p* = 0.378) nor the *test number of first-released bee* in the delay tests (GLMM: 95% CI = 1.21 (0.66–2.23), *p* = 0.529) had a significant impact on the time taken to first push on the block. Furthermore, the likelihood of the first-released bee to push after the second-released bee was higher for partnered bees than single controls (model 2; GLMM: 95% CI = 3.11 (0.74–5.48), *p* = 0.010) (figure 2*b*). Neither the *experiment* (GLMM: 95% CI = 1.18 (−0.69 to 3.05), *p* = 0.215) nor the *test number of first-released bee* in the delay tests (GLMM: 95% CI = 0.39 (−1.08 to 1.87), *p* = 0.600) had a significant impact on the likelihood of the first-released bee to push after the second-released bee. The proportion of first pushes by first-released bees that occurred when the second-released bee was present was also greater for partnered bees than single controls (model 3; permutation test: 95% CI = 24.39 (−1.30 to 1.24), *p* < 0.001; figure 2*c*). Single control’s first push never occurred when the second-released bee was present.

### (c) Study 2. Tunnel task

#### (i) Methods for tunnel task

**Figure 3.**
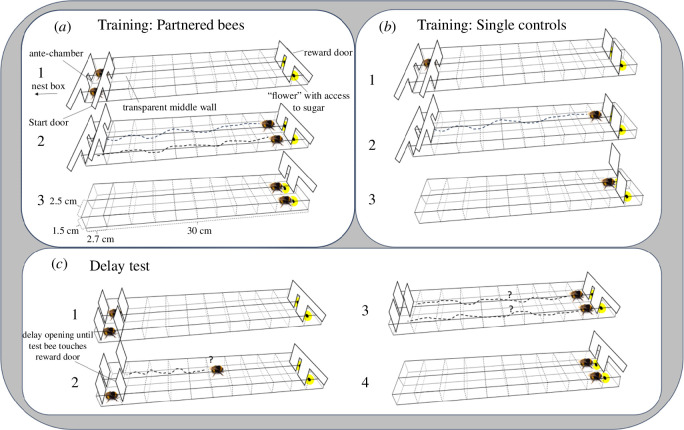
Tunnel task. Training and testing methods used for the partnered bees and single controls. (*a*) Just prior to the start of a bout, partnered bees were isolated in the antechamber, at which point the start door was removed allowing both bees simultaneous access to the tunnels (1). Bees then walked through the transparent tunnel and when they both touched the reward door at the other end simultaneously (2), the reward door was removed by the experimenter which gave simultaneous access to the sugar water via a hole in the tunnel floor (3). (*b*) Single controls were trained in a similar manner, just without a partner bee. (*c*) In the delay test, test bees in both groups had a partner in the antechamber (1). The partner was released only after the test bee touched the door (2). Bees gained access to the reward after touching the reward door simultaneously (3).

Bumblebees’ natural behaviour to fly and explore within the block-pushing task made it difficult to assess more than the straightforward effect of a partner’s presence in the delay tests. To explore deeper into the potential of bumblebees’ cooperative behaviour being socially influenced and potentially actively coordinated, we devised an experimental task for bumblebees inspired by the work of Jaakkola *et al*. [29]. In their study, bottlenose dolphins engaged in a button-pressing task that demanded behavioural synchronization. Here, we adapted the dolphin paradigm to suit bumblebees so that we could measure several parameters over training and delay tests. Bees were required to walk through a transparent 30 cm long tunnel and touch a door at the other end of the tunnel either alone (single control group) or with their partner located in an adjacent tunnel (partnered group; figure 3) to get access to a flower containing sugar water.

The partnered group, 26 bees from three different colonies (2, 12, 12), underwent a training phase consisting of 10 bouts. The first bout was considered pre-training wherein the bees learned the location of the reward at the other end of the tunnel and was excluded from the analysis. Just prior to the start of each bout, the partnered bees were isolated in the antechamber, one in each tunnel (figure 3*a*1). The experimenter removed the start door giving both bees simultaneous access to the tunnels (figure 3*a*1; electronic supplementary material, video S4). The experimenter removed the reward door at the other end of the tunnel when both bees touched the reward door simultaneously (figure 3*a*2). A hole (Ø = 1 mm) in the tunnel floor allowed the bees to access a container with 40% sugar water. A yellow circle (Ø = 9 mm) was painted around the hole to facilitate the bees locating the reward (figure 3*a*3). The purpose of the training phase was to familiarize the bees with the task and establish coordination between them.

The single control group consisted of seven bees from two colonies (5, 2), and they were individually trained to touch the reward door to access the rewarding flower (figure 3*b*). These bees underwent a training phase comprising 10 bouts. Similar to the partnered group, the first bout for the single control group was considered pre-training and excluded from analysis.

Following the training phase, partnered bees’ behaviour was assessed over two delay tests. In the delay tests, one bee was let into the tunnel (first-released), and then the other bee (second-released) was let into the tunnel only after the first-released bee had touched the reward door (figure 3*c*; electronic supplementary material, video S5). The reason for two delay tests was that each bee experienced both being released first and second. The delay test (first or second) in which a bee acted as a first-released bee was randomized. For the single control group, each bee underwent one delay test wherein she was confined in the antechamber with a pre-trained bee from the partnered group and the same colony in the adjacent antechamber. The single control bee was released into the tunnel alone and let through the reward door once she touched it.

All bouts were recorded using an iPhone 8 (Apple Inc., Cupertino, CA) placed above the tunnels. Behavioural measurements were obtained through video analysis and are described below. Similar to the block-pushing experiments, the tunnel experiment video filenames were labelled by a non-coder such that the coder was at least initially blind to the group identity for the delay tests (two bees in each group). The videos were analysed by O.J.L. (100%) and K.M. (5%). ICC analysis was employed to assess the agreement among the two observers for the measured variables. The mean ICC value was 0.91 (95% CI: 0.81–1), indicating a high level of agreement among the coders. For the statistical analyses, we employed GLMMs using the glmmTMB function from the R package (v. 4.3.0) [41] glmmTMB (v. 1.1.8) [42]. GLMMs were applied for the tunnel task (models 4–7) as described below.

Model 4 assessed whether, in the partnered group, the first-released bees took longer to reach the reward door in the delay test compared with their last three training bouts, and relative to the single control group. We hypothesized that if partnered bees’ cooperative behaviour was socially influenced, they should slow down or hesitate in the delay tests because of their partner’s delayed release. In contrast, bees in the single control group should have no reason to slow down. We used the *time taken to touch the door* as the response variable. We first analysed all datasets from training and delay tests. To avoid the variation and complexity of bees’ turning behaviours, and to focus on whether bees slowed down when their partner was delayed, we then analysed these data excluding bouts in which bees turned around prior to reaching the door. The explanatory variables included the interaction between the *phase* (last three training bouts or delay test) and the *group* (partnered bees or single controls). *Colony/bee* was used as a random variable. A Gaussian distribution was used as the family.

In model 5, we examined whether the second-released bees took less time from release to reach the reward door in the delay test compared with the first-released bees. If bees’ coordinated efforts in the tunnel task were socially influenced, then we should expect that, because their partner was not released at the same time, the first-released bees should hesitate and take longer to reach the door in the delay test. In contrast, the second-released bees should realize their partner had already entered the tunnel, and therefore should have no reason to slow down, but instead try to catch up to their partner. The *time taken from the release to touching the door* was used as the response variable. The explanatory variable was the *test number of first-released bee* (whether the individual bee acted as a first-released bee in the first or second delay test). *Colony/bee* was used as a random variable. A Gaussian distribution was used as the family.

In model 6, we assessed whether the single control bees were just as quick to reach the reward door in the delay test compared with the second-released bees in the partnered group. Similar to the second-released partnered bees, the single control bees should have no reason to slow down. Therefore, we expected that the mean time to reach the door would not differ significantly between single control bees and second-released partnered bees. The *time taken from the release to touching the door* was used as the response variable. The explanatory variable was the *group* (single controls or second-released partnered bees). *Colony/bee* was used as a random variable. A Gaussian distribution was used as the family.

In model 7, we tested the likelihood of bees to turn around before reaching the door. We hypothesized that if bees had a basic understanding of the coordinated aspect of the task, they should realize their partner was not released into the tunnel and, before arriving at the reward door, be more likely to turn around in search of their partner with the aim of facilitating coordinated action. Otherwise, bees should have no reason to turn around before first reaching the door. We calculated the likelihood by determining the number of bees that turned before reaching the door and dividing this by the total number of bees in the dataset. To estimate the precision of our likelihood estimate, we computed a 95% CI using the Wilson method. A binomial distribution was used as the family.

In model 8, we examined bees’ orientation within 1 s of them simultaneously pushing at the door, and gaining access to the reward. The question was whether partnered bees, like other animals exhibiting socially influenced cooperative behaviour [14,43] had a tendency to look in their partner’s direction while performing the cooperative task. Although bees have a near 360° field of view, like most animals they tend to turn their body axis towards stimuli of interest [44]. The two stimuli of interest in this cooperative task were the door, which blocked access to the reward, and their partner, with whom they simultaneously needed to push the door. Therefore, we hypothesized that if bees’ cooperative actions are socially influenced, we should expect more instances of body orientation angled at least slightly towards the adjacent tunnel. If there was no social influence, bees should be focused only on the reward door, and thus be just as likely to face towards the adjacent tunnel as away from it, and perhaps more likely to just face straight ahead. As a very conservative measure, we chose to assign a value of 1 to a bee, i.e. designate their orientation as towards the adjacent tunnel, only if both their antennae had touched the middle wall (See electronic supplementary material, video S6 for examples). Otherwise, we assigned a value of zero. This method significantly reduced any chance for bias by the encoder and ensured that the bee was clearly angled towards their partner. As a further conservative measure, we chose 0.5 as the chance level. The binary response variable was *whether the bee was oriented towards the adjacent tunnel or not*. The explanatory variable was the *group* (partnered bees or single controls). *Colony/bee* was used as a random variable. A binomial distribution with a logarithmic link function was used as the family.

In model 9, we assessed the probability of partnered bees, when heading away from the reward door, to reverse their direction back towards the reward door when encountering their partner directly beside them in the adjacent tunnel. We hypothesized that if bees understood the role of their partner, those bees heading away from the reward door might be looking for their partner. In turn, they should notice the presence and travel direction of their partner once they were directly across from each other, and quickly turn around in an attempt to coordinate their actions at the reward door. If bees were acting entirely individually, then when one bee happens to turn around, the likelihood of their partner being in any section of the tunnel should be equal. We therefore divided the tunnel into 10 equal sections, 2.7 cm each, and recorded which section each bee was in during each bee’s turn. The response variable for the model was the *proportion of instances in which a partnered bee reversed their direction towards the rewarding door when beside their partner,* in training and the delay tests. A value of 0 indicates all turns by one bee in a bout occurred when their partner was not in the section directly across from them. A value of 1 signifies all turns by one bee in a bout occurred when their partner was in the section directly across from them (see electronic supplementary material, video S7 for examples). Additionally, we incorporated weighting in the model to account for the number of turns within the tunnel. To establish a baseline, the offset function was used, setting the chance level to 0.1, which corresponded to the demarcated 10 sections of the tunnel, resulting in a 10% likelihood of making a turn in the same section as the partner by random chance. *Colony/bee* was used as a random variable. A binomial distribution with a logarithmic link function was used as the family.

In model 10, we used a binomial test to explore whether turns that occurred when both bees were in the same section of the tunnel resulted in both bees heading towards or away from the reward door. The Wilson method was employed to calculate the 95% CI for the ratio of instances where bees turned away. If bees had no understanding of their partner’s role in the task but were still socially influenced by their partner’s presence and direction of travel, we would expect bees to be as likely to turn in the wrong direction as the right direction in the above (model 9) situations. In contrast, if bees had a basic understanding of their partner’s role in the task, then when encountering each other in the same section and heading in the opposite direction, the bee heading away from the door should be more likely to turn around than the bee already heading towards the door. A binomial distribution was used as the family.

#### (ii) Results for tunnel task

**Figure 4.**
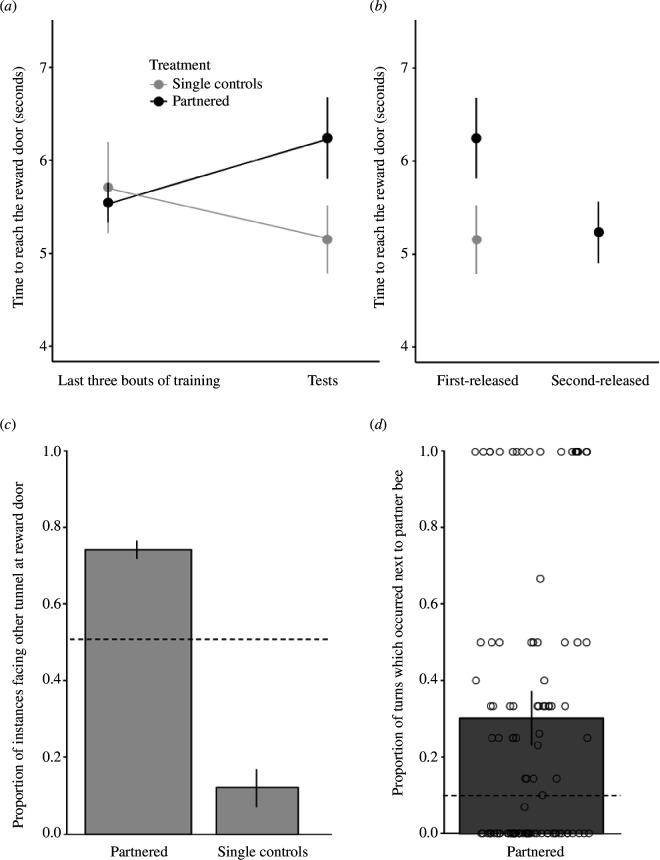
Results of the tunnel task. (*a*) Time for bees to reach the reward door during the last three bouts of training and delay tests. (*b*) Differences in the time to reach the reward door for partnered bees (first- and second-released) and single controls (all first-released) during the delay test. (*c*) Proportions of instances when a bee at the reward door faced the adjacent tunnel just prior to simultaneously pushing and being left through the door. The dashed horizontal line at 0.50 represents a conservatively set chance likelihood that a bee would face the adjacent tunnel while at the reward door (§2; electronic supplementary material, video S6). (*d*) Mean proportion of partnered bees turning back toward the reward door when their partner was located directly across in the adjacent tunnel. Open circles depict individual samples encompassing both the training bouts and the delay tests. A value of 0 indicates all turns by one bee in a bout occurred when their partner was not in the section directly across from them. A value of 1 signifies all turns by one bee in a bout occurred when their partner was in the section directly across from them. The dashed horizontal line at 0.1 represents chance level (§2). Bars and circles indicate mean values, and vertical lines represent s.e.m. in all figures.

An initial analysis showed no significant difference for the first-released bees in the partnered group in the amount of time to reach the reward door between the end of training and the delay test (GLMM: 95% CI = 8.22 (−6.06 to 22.49), *p* = 0.259). However, we noticed that first-released bees often turned around prior to first reaching the reward door. With the aim of comparing just the time taken to traverse the tunnel and push the door, without considering the additional complexity of turning behaviours, we repeated the analysis while excluding those bouts in which a turn occurred before first reaching the door. We then found that during the delay tests, first-released bees in the partnered group took longer to reach the reward door compared with the end of training (GLMM: 95% CI = 0.77 (0.07–1.47), *p* = 0.03) (figure 4*a*; slope of black line). There was no significant difference in time taken to reach the reward door during the end of training between the first-released bees in the partnered and single control groups (GLMM: 95% CI = −0.12 (−1.29 to 1.05), *p* = 0.843; figure 4*a*, left side). However, from the end of training to the delay test, the first-released bees in the partnered group showed an increase in the time taken to reach the reward door in comparison with the bees in the single control group (GLMM: 95% CI = 1.40 (0.06–2.74), *p* = 0.04; figure 4*a*; compare positive slope of black line with negative slope of grey line). Furthermore, for the partnered group, the second-released bees took significantly less time to reach the reward door during the delay test compared with the first-released bees (GLMM: 95% CI = −1.05 (−1.64 to −0.45), *p* < 0.001; figure 4*b*). In contrast, single control bees (only released first) were just as quick to reach the reward door as second-released bees in the partnered group (GLMM: 95% CI = −0.28 (−1.64 to 1.07), *p* = 0.681; figure 4*b*).

With regard to the observed turning behaviours, the likelihood of first-released bees in the partnered group turning around before they first reached the reward door was 0.33 (95% CI: 0.11–0.60), while single control bees never turned prior to reaching the door. From the point at which the first-released bee in the partnered group first touched the door and turned around to the point when both bees simultaneously touched the door, the first-released bee spent 50% of this time away from the door (95% CI: 0.46–0.54). We also found that, just before simultaneously pushing at and being let through the door, first-released bees in the partnered group were more likely to be oriented towards the adjacent tunnel compared with single controls (GLMM: 95% CI = −4.45 (−5.84 to −3.05), *p* <0.001; figure 4*c*). After reaching the door, first-released bees in the partnered group that had turned around and were heading away from the reward door were more likely, compared with chance, to turn back towards the reward door if they encountered their partner directly across in the adjacent tunnel (GLMM: 95% CI (back-transformed from the log scale) = 2.72 (1.97–3.75), *p* < 0.001; figure 4*d*). Considering all the instances in which a bee turned when both bees were in the same section of the tunnel and heading in the opposite direction, this scenario was much more likely to result in both bees heading towards the reward door (binomial test: 95% = 0.99 (0.93–1), *p* < 0.001). Specifically, in 73 of the above-described instances (among 22 out of 25 bees), the bee heading away from the door turned around, whereas only once did the bee heading towards the door change direction.

## 3. Discussion

Our study shows that coordination in the two tasks we examined is not merely a by-product of bees’ individual efforts. First-released bees in the partnered group tended to take longer to first push on the block or reach the reward door in the delay tests compared with single control bees (both paradigms), with their delayed partner (both paradigms) and with training (tunnel task). First-released bees in the partnered group were less likely to push the block before the second-released bee and more likely to first push the block when the second-released bee was present, compared with single controls. Furthermore, just before simultaneously pushing at the door, first-released bees in the partnered group were more likely to have their bodies oriented towards the adjacent tunnel compared with the single controls. Together, these results show that bumblebees waited for their partner and attended to their partner’s presence/absence at the point of cooperation, and thus strongly indicate that their coordination in both tasks was socially influenced.

With regard to whether bees actively coordinated in the tasks, a methodological difference between our tunnel task design and the dolphin study that helped inspire it [29], is a noteworthy first point of consideration. Dolphins were required to synchronize when they first touched the reward buttons in order to obtain a reward. Dolphins’ ability to precisely synchronize their behaviour gave strong support to the idea that their cooperation was actively coordinated. In contrast, bumblebees were not required to precisely synchronize their first push, but rather only needed to eventually synchronize any of their individual pushes within each training bout or test. Therefore, in theory, even if bees’ coordination was socially influenced, once the first-released bee reached the reward door they could have simply continued to push until the other bee eventually arrived at the door. However, after first reaching the door in the delay test, most of the first-released bees in the partnered group (18/26) did not remain at the door until their partner arrived. Rather, they spent only a few seconds at the door before turning around and, prior to eventually simultaneously pushing with their partner, spent half of the subsequent time away from the door.

Importantly, we used other measured behaviours to examine whether bees may have been actively coordinating their efforts. If bees’ coordination went beyond social influence, we should have observed attempts to somehow facilitate coordination. The likelihood of a first-released bee in the partnered group to turn around before reaching the door was 33%, whereas single control bees never turned prior to reaching the door. First-released bees in the partnered group walking away from the reward door were more likely, compared with chance, to turn back towards the reward door when they encountered their partner walking towards the door directly across in the adjacent tunnel. This turning scenario occurred 73 times resulting in both bees heading towards the reward door and only once resulting in bees heading in the wrong direction. We argue that these results suggest the possibility of an intentional strategy for temporal and spatial coordination and to facilitate coordination. We believe these observations may give some support for attempts at active coordination in its simplest form, i.e. a basic understanding of the coordinated requirements of the task. However, these types of measurements could also be interpreted as responses to the presence, absence and direction of social information without the intent to actively facilitate coordination (i.e. socially influenced cooperation). Rather than turning behaviours indicating bees attempt to recruit or coordinate with the other bee, turning in the observed scenarios may have been because the bees associated the presence of another bee at the door with access to reward, and adjusted their behaviour in these different situations accordingly. Further investigations will help confirm, clarify and build on our current results and interpretations (cf. [29]). Designs that require precise synchronization, provide individual and cooperative task options, and allow for and can measure potential recruitment behaviours would be particularly valuable in testing whether bees’ behaviours are truly flexible and a result of active coordination.

Considering the evolution of coordination, it is intriguing to consider why bumblebees might have developed this capacity. Bumblebees appear to operate alone while foraging in the wild and may at times avoid other foraging bumblebees [31]. However, it is possible that the capacity for coordinated behaviours and a basic understanding of a potential partner’s role during coordinated effort is widespread across the animal kingdom simply because it has conferred selective advantages for enhancing resource acquisition, predator avoidance or other key aspects of survival. It may also be that the capacities for socially influenced cooperation and (possibly) actively coordinated collaboration evolved in bumblebees in response to the pressures faced within the hive rather than while foraging. This domain general ability may then be coopted in novel situations when required to by certain environmental pressures, e.g. novel food sources or odd paradigms provided by researchers.

Looking ahead, our study opens the door to future investigations into whether bumblebees, and other invertebrates, possess a more nuanced understanding of their partner’s role in cooperative tasks, including more evidence for or against actively coordinated collaboration. A further question is whether bumblebees might show any evidence of what Duguid and Melis [5] consider ‘true collaboration’, i.e. *collaboration based on shared intentionality*. Duguid and Melis argue that chimpanzees, and potentially other great apes, cooperate with shared intentionality. A clearer comparative understanding of cooperation, including the possibility of shared intentionality in other more distantly related species, will require an expansion of experimental methods and measurement techniques with new levels of accuracy and accessibility, such as high-speed video recording and deep learning-based multi-animal pose tracking [45]. These technologies could offer a more detailed and accurate analysis of animal behaviours during collaborative tasks, capturing subtle cues or gestures—e.g. whether individuals employ strategies to attract or direct their partners towards the task—that might go unnoticed with traditional methods. This approach could provide a comprehensive picture of the mechanisms underlying coordination and potentially reveal new forms of active cooperation. As shown here with our work, the categories proposed by Duguid and Melis [5] provide a framework to probe whether behaviours in cooperative tasks represent individual efforts, a simple recognition of partner presence or a deeper understanding of roles and goals. This line of research promises exciting avenues for understanding the intricacies of social behaviour in bumblebees and other species, ultimately contributing to a more nuanced comprehension of the underlying mechanisms driving cooperation in different animals.

## Data Availability

Data available from the Dryad Digital Repository [46]. Electronic supplementary material is available online [47].

## References

[1] Melis AP , Semmann D . 2010 How is human cooperation different? Phil. Trans. R. Soc. B **365** , 2663–2674. (10.1098/rstb.2010.0157) 20679110 PMC2936178

[2] Boyd R , Richerson PJ . 1988 Culture and the evolutionary process. Chicago, IL: University of Chicago Press.

[3] Patzelt A , Kopp GH , Ndao I , Kalbitzer U , Zinner D , Fischer J . 2014 Male tolerance and male–male bonds in a multilevel primate society. Proc. Natl Acad. Sci. USA **111** , 14740–14745. (10.1073/pnas.1405811111) 25201960 PMC4205614

[4] Connor RC , Heithaus MR , Barre LM . 1999 Superalliance of bottlenose dolphins. Nature **397** , 571–572. (10.1038/17501)

[5] Duguid S , Melis AP . 2020 How animals collaborate: underlying proximate mechanisms. Wiley Interdiscip. Rev. Cogn. Sci. **11** , e1529. (10.1002/wcs.1529) 32342659

[6] Bshary R , Grutter AS . 2006 Image scoring and cooperation in a cleaner fish mutualism. Nature **441** , 975–978. (10.1038/nature04755) 16791194

[7] Hölldobler B , Wilson EO . 2008 The superorganism: the beauty, elegance, and strangeness of insect societies, 1st edn. New York, NY: W. W. Norton & Company.

[8] Dugatkin LA . 1997 Cooperation among animals: an evolutionary perspective. Oxford, UK: Oxford University Press. See https://academic.oup.com/book/54123.

[9] Crawford MP . 1937 The cooperative solving of problems by young chimpanzees. Comp. Psychol. Monogr. **14** , 1–88.

[10] Seed AM , Clayton NS , Emery NJ . 2008 Cooperative problem solving in rooks (Corvus frugilegus). Proc. R. Soc. B **275** , 1421–1429. (10.1098/rspb.2008.0111) PMC2602707 18364318

[11] Visalberghi E , Quarantotti BP , Tranchida F . 2000 Solving a cooperation task without taking into account the partner’s behavior: the case of capuchin monkeys (Cebus apella). J. Comp. Psychol. **114** , 297–301. (10.1037/0735-7036.114.3.297) 10994846

[12] Suchak M , Eppley TM , Campbell MW , de Waal FBM . 2014 Ape duos and trios: spontaneous cooperation with free partner choice in chimpanzees. Peer J. **2** , e417. (10.7717/peerj.417) 24949236 PMC4060033

[13] Chalmeau R , Gallo A . 1996 What chimpanzees (Pan troglodytes) learn in a cooperative task. Primates **37** , 39–47. (10.1007/BF02382918)

[14] Mendres KA , de Waal FBM . 2000 Capuchins do cooperate: the advantage of an intuitive task. Anim. Behav. **60** , 523–529. (10.1006/anbe.2000.1512) 11032655

[15] Drea CM , Carter AN . 2009 Cooperative problem solving in a social carnivore. Anim. Behav. **78** , 967–977. (10.1016/j.anbehav.2009.06.030)

[16] Hirata S , Fuwa K . 2007 Chimpanzees (Pan troglodytes) learn to act with other individuals in a cooperative task. Primates **48** , 13–21. (10.1007/s10329-006-0022-1) 17103081

[17] Plotnik JM , Lair R , Suphachoksahakun W , de Waal FBM . 2011 Elephants know when they need a helping trunk in a cooperative task. Proc. Natl Acad. Sci. USA **108** , 5116–5121. (10.1073/pnas.1101765108) 21383191 PMC3064331

[18] Marshall-Pescini S , Schwarz JFL , Kostelnik I , Virányi Z , Range F . 2017 Importance of a species’ socioecology: wolves outperform dogs in a conspecific cooperation task. Proc. Natl Acad. Sci. USA **114** , 11 793–11 798. (10.1073/pnas.1709027114) 29078337 PMC5676910

[19] Heaney M , Gray RD , Taylor AH . 2017 Keas perform similarly to chimpanzees and elephants when solving collaborative tasks. PLoS one **12** , e0169799. (10.1371/journal.pone.0169799) 28199322 PMC5310852

[20] Melis AP , Hare B , Tomasello M . 2006 Chimpanzees recruit the best collaborators. Science **311** , 1297–1300. (10.1126/science.1123007) 16513985

[21] Chalmeau R . 1994 Do chimpanzees cooperate in a learning task? Primates **35** , 385–392. (10.1007/BF02382735)

[22] Chalmeau R , Lardeux K , Brandibas P , Gallo A . 1997 Cooperative problem solving by orangutans (Pongo pygmaeus). Int. J. Primatol. **18** , 23–32. (10.1023/A:1026337006136)

[23] Schmelz M , Duguid S , Bohn M , Völter CJ . 2017 Cooperative problem solving in giant otters (Pteronura brasiliensis) and Asian small-clawed otters (Aonyx cinerea). Anim. Cogn. **20** , 1107–1114. (10.1007/s10071-017-1126-2) 28840405 PMC5640742

[24] Massen JJM , Ritter C , Bugnyar T . 2015 Tolerance and reward equity predict cooperation in ravens (Corvus corax). Sci. Rep. **5** , 15021. (10.1038/srep15021) 26442633 PMC4595729

[25] Péron F , Rat-Fischer L , Lalot M , Nagle L , Bovet D . 2011 Cooperative problem solving in African grey parrots (Psittacus erithacus). Anim. Cogn. **14** , 545–553. (10.1007/s10071-011-0389-2) 21384141

[26] Ostojić L , Clayton NS . 2014 Behavioural coordination of dogs in a cooperative problem-solving task with a conspecific and a human partner. Anim. Cogn. **17** , 445–459. (10.1007/s10071-013-0676-1) 23995845 PMC3920030

[27] Tassin de Montaigu C , Durdevic K , Brucks D , Krasheninnikova A , von Bayern A . 2020 Blue‐throated macaws (Ara glaucogularis) succeed in a cooperative task without coordinating their actions. Ethology **126** , 267–277. (10.1111/eth.12973)

[28] Suchak M , Watzek J , Quarles LF , de Waal FBM . 2018 Novice chimpanzees cooperate successfully in the presence of experts, but may have limited understanding of the task. Anim. Cogn. **21** , 87–98. (10.1007/s10071-017-1142-2) 29147914

[29] Jaakkola K , Guarino E , Donegan K , King SL . 2018 Bottlenose dolphins can understand their partner’s role in a cooperative task. Proc. R. Soc. B **285** , 20180948. (10.1098/rspb.2018.0948) PMC6170804 30232161

[30] Melis AP , Raihani NJ . 2023 The cognitive challenges of cooperation in human and nonhuman animals. Nat. Rev. Psychol. **2** , 523–536. (10.1038/s44159-023-00207-7)

[31] Kawaguchi LG , Ohashi K , Toquenaga Y . 2007 Contrasting responses of bumble bees to feeding conspecifics on their familiar and unfamiliar flowers. Proc. R. Soc. B **274** , 2661–2667. (10.1098/rspb.2007.0860) PMC2279218 17698483

[32] Pleasants JM . 1981 Bumblebee response to variation in nectar availability. Ecology **62** , 1648–1661. (10.2307/1941519)

[33] Brosnan SF , Salwiczek L , Bshary R . 2010 The interplay of cognition and cooperation. Phil. Trans. R. Soc. B **365** , 2699–2710. (10.1098/rstb.2010.0154) 20679113 PMC2936177

[34] Alem S , Perry CJ , Zhu X , Loukola OJ , Ingraham T , Søvik E , Chittka L . 2016 Associative mechanisms allow for social learning and cultural transmission of string pulling in an insect. PLoS Biol. **14** , e1002564. (10.1371/journal.pbio.1002564) 27701411 PMC5049772

[35] Loukola OJ , Solvi C , Coscos L , Chittka L . 2017 Bumblebees show cognitive flexibility by improving on an observed complex behavior. Science **355** , 833–836. (10.1126/science.aag2360) 28232576

[36] Ravi S , Siesenop T , Bertrand O , Li L , Doussot C , Warren WH , Combes SA , Egelhaaf M . 2020 Bumblebees perceive the spatial layout of their environment in relation to their body size and form to minimize inflight collisions. Proc. Natl Acad. Sci. USA **117** , 31494–31499. (10.1073/pnas.2016872117) 33229535 PMC7733852

[37] Galpayage Dona HS , Solvi C , Kowalewska A , Mäkelä K , MaBouDi H , Chittka L . 2022 Do bumble bees play? Anim. Behav. **194** , 239–251. (10.1016/j.anbehav.2022.08.013)

[38] Solvi C , Baciadonna L , Chittka L . 2016 Unexpected rewards induce dopamine-dependent positive emotion-like state changes in bumblebees. Science **353** , 1529–1531. (10.1126/science.aaf4454) 27708101

[39] Gamer M , Lemon J , Singh IFP . 2019 Irr: various coefficients of interrater reliability and agreement. See https://cran.r-project.org/web/packages/irr/irr.pdf

[40] Brooks ME , Kristensen K , van Benthem K , Magnusson A , Berg CW , Nielsen A , Skaug HJ , Mächler M , Bolker BM . glmmTMB balances speed and flexibility among packages for zero-inflated generalized linear mixed modeling. R J. **9** , 378. (10.32614/RJ-2017-066)

[41] R Core Team . 2011 *R: A language and environment for statistical computing*. Vienna, Austria: R Foundation for Statistical Computing.

[42] Brooks M , *et al* . 2023 glmmTMB: generalized linear mixed models using template model builder. See https://cran.r-project.org/web/packages/glmmTMB/glmmTMB.pdf.

[43] Hattori Y , Kuroshima H , Fujita K . 2005 Cooperative problem solving by tufted capuchin monkeys (Cebus apella): spontaneous division of labor, communication, and reciprocal altruism. J. Comp. Psychol. **119** , 335–342. (10.1037/0735-7036.119.3.335) 16131262

[44] Paulk AC , Stacey JA , Pearson TWJ , Taylor GJ , Moore RJD , Srinivasan MV , van Swinderen B . 2014 Selective attention in the honeybee optic lobes precedes behavioral choices. Proc. Natl Acad. Sci. USA **111** , 5006–5011. (10.1073/pnas.1323297111) 24639490 PMC3977245

[45] Mathis MW , Mathis A . 2020 Deep learning tools for the measurement of animal behavior in neuroscience. Curr. Opin. Neurobiol. **60** , 1–11. (10.1016/j.conb.2019.10.008) 31791006

[46] Loukola OJ , Antinoja A , Mäkelä K , Arppi J , Peng F , Solvi C . 2024 Evidence for socially influenced and potentially actively coordinated cooperation by bumblebees. Dryad (10.5061/dryad.gtht76htr) PMC11061644 38689557

[47] Loukola OJ , Mäkelä K , Arppi J , Antinoja A , Peng F , Solvi C . 2024 Supplementary material from: Evidence for socially influenced and potentially actively coordinated cooperation by bumblebees. Figshare (10.6084/m9.figshare.c.7162538) PMC11061644 38689557

